# Limited Physical Functioning in United States Adults with Arthritis: Findings from the 2021 Medical Expenditure Panel Survey

**DOI:** 10.3390/diseases12080170

**Published:** 2024-08-01

**Authors:** David R. Axon

**Affiliations:** Department of Pharmacy Practice & Science, R. Ken Coit College of Pharmacy, The University of Arizona, 1295 N. Martin Ave., Tucson, AZ 85721, USA; draxon@arizona.edu; Tel.: +1-520-621-9561

**Keywords:** arthritis, physical functioning, United States adults

## Abstract

There is little published research on limited physical functioning in United States (US) adults with arthritis. The objective of this cross-sectional 2021 Medical Expenditure Panel Survey (MEPS) database study was to investigate the variables associated with limited physical functioning in US adults with arthritis. Logistic regression tested the associations of predisposing, enabling, and need variables with the dependent variable (limited physical functioning). This study included 5102 US adults with arthritis, reflecting an estimated weighted population of 64,136,870 US adults with arthritis. In the final multivariable logistic regression model, age ≥ 70 and ages 60–69 (vs. 18–49 years), female (vs. male) sex, having quite a bit/extreme or moderate (vs. little) pain, and having 6+ or 4–5 (vs. 0–1) comorbid conditions were all associated with higher odds of the person stating they had limited physical functioning. Whereas high school or less (vs. more than high school), being employed (vs. unemployed), being married (vs. not married), having excellent/very good or good (vs. poor) general health, and exercise (vs. no exercise) were each associated with lower odds of the person reporting they had limited physical functioning. Future work may be considered to explore these variables in greater detail.

## 1. Introduction

Limited physical functioning may be defined on the basis of the level of difficulty a person has with various functioning domains. Several working definitions exist. The Centers for Disease Control and Prevention (CDC) defines a person with limited physical functioning as someone who has a lot of difficulty or who cannot carry out any one of the following core realms: self-care, seeing, mobility, hearing, communication, and cognition [1]. National surveys in the United States (US), such as the National Health Interview Survey (NHIS) and the Medical Expenditure Panel Survey (MEPS), facilitated by the Agency for Healthcare Research and Quality (AHRQ), have their own working definitions for limited physical functioning [1,2]. For instance, MEPS defines a person as having limited physical functioning if they find it difficult to walk, climb the stairs, grasp objects, reach above their head, lift, bend, stoop, or stand for extended time periods [2]. Limited physical functioning may arise as a consequence of physical or mental impairment and can be considered a disability [1]. Limited physical functioning can affect a person’s life opportunities through poorer educational and employment attainment [3,4,5]. A contemporary study of community-dwelling older US adults found that limited physical functioning was associated with a higher probability of participating in activities related to poor health, such as watching television and having medical appointments, as well as a lower likelihood of participating in physical activities, household chores, or social events [6]. One cross-sectional study using 2017 MEPS data found that approximately 39% of US adults over the age of 50 with some degree of pain had limited physical functioning [7], while another cross-sectional analysis using the 2020 MEPS reported that the prevalence of limited physical functioning was 56% among US older adults with pain who used opiates [8].

Arthritis is a prevalent chronic condition experienced by over 53 million adults (or 19% of adults) in the US in recent years (2019–2022) [9,10]. The increase in arthritis cases is expected to be over 78 million adults (or 26% of adults) in the US by 2040, which is further cause for concern [11]. Arthritis is characterized as a typically incapacitating disease with symptoms of inflammation, pain, redness, reduced motion, stiffness, swelling, and/or tenderness of predominantly body joints [12]. The two main types of arthritis, rheumatoid arthritis (an autoimmune disorder affecting joints and other tissues) and osteoarthritis (degenerative breakdown of joints and joint tissue), account for the majority of cases, although there are many other forms of arthritis that are less common [13]. While arthritis can occur at any age, it is most prevalent among older adults, with approximately 54% of adults aged ≥75 years having arthritis and 44% of adults aged 65–74 years having arthritis [14]. However, arthritis is increasingly occurring in younger adults, with approximately 4% of adults aged 18–34 having arthritis, 12% of adults aged 35–49 years having arthritis, and 29% of adults aged 50–64 years having arthritis [14]. Arthritis occurs more frequently in women and can be influenced by a multitude of demographic attributes that are not always well understood, such as race/ethnicity, education, wealth, metropolitan status, and national region [9,10]. Data from the 1993 Asset and Health Dynamic Survey Among the Oldest Old found the occurrence of arthritis among older US adults varied by race/ethnicity, at 25% among non-Hispanic whites, 40% among non-Hispanic blacks, and 44% among Hispanics [15]. Among US adults aged ≥ 18 years, data from the NHIS found that approximately 21% of non-Hispanic whites had arthritis, 19% of non-Hispanic blacks had arthritis, 15% of Hispanics had arthritis, and 11% of Asians had arthritis [14]. Individuals are at higher risk of arthritis if they are older or female, if their parents or siblings have arthritis, if they have previously had an injury that affected a joint(s), and if they are overweight or obese [12,16]. Arthritis is a major predictor of disabilities such as difficulty walking or ascending stairs, completing daily domestic duties, and overall limited physical functioning [17]. Beyond physical disabilities, arthritis can adversely affect one’s mental health and overall life quality [18,19]. Adults with arthritis find it costly to manage their condition [20,21,22]. For example, in 2013, it accounted for over $300 billion in healthcare expenditure and lost earnings [22]. Arthritis is typically managed by relieving symptoms and improving joint function, which can involve a personalized regimen that consists of medications, physical therapy, and/or joint repair or replacement surgery. Medications can include non-steroidal anti-inflammatory drugs (NSAIDs), either as a solid oral dosage form or a cream/gel; counterirritants (creams/ointments that are thought to interfere with the transmission of pain); corticosteroids (either as a solid oral dosage form or an injection); and disease-modifying antirheumatic drugs (DMARDs). Side effects of these medications include stomach irritation, bone thinning, weight gain, and an increased risk of cardiovascular conditions and infections [23]. Adults with pain, such as that caused by arthritis, are often burdened with the need for multiple medications and other management strategies [24].

Given the prevalence and negative consequences of both limited physical functioning and arthritis in the US, there is interest in examining the factors associated with limited physical functioning specifically in US adults with arthritis. However, there is little contemporary research on limited physical functioning among US adults with arthritis. One cohort of older adults in 1998 to 2000 identified several risk factors for limited physical functioning; the most prominent was a lack of frequent physical activity, while others included advanced age, reduced cognition, depression, diabetes, physical limitations, lack of alcohol consumption, stroke, and reduced vision [25]. With the aim of addressing this knowledge gap, this study’s objective was to investigate the variables associated with limited physical functioning among US adults with arthritis.

## 2. Materials and Methods

This cross-sectional MEPS database study utilized the MEPS 2021 full-year consolidated data file (28,336 individuals). Briefly, MEPS is an ongoing nationwide survey conducted on behalf of AHRQ in the US that surveys a nationally representative sample of households. Households are selected for participation in MEPS using the NHIS sampling framework. MEPS interviewers contact a member of the household and ask them to provide a voluminous amount of healthcare-related data throughout multiple panels or rounds of interviews over 2 years. These include data on healthcare expenditure and other financial considerations, chronic and common health conditions (e.g., arthritis), and health states including various disabilities (e.g., limited physical functioning). MEPS staff check the quality of these data and supplement them as necessary from data provided by medical providers and insurance providers. The data are collated, coded, and later made publicly available for researchers to download and use at no financial cost [26].

MEPS subjects were included in the current study if they were alive for the entirety of 2021, 18 years of age or older, and had arthritis. The dependent variable in this study was whether (yes or no) an individual reported having limited physical functioning. Limited physical functioning was indicated if an individual found it difficult to walk, climb the stairs, grasp objects, reach above their head, lift, bend, stoop, or stand for extended time periods [2,27]. The independent variables in this study were categorized using the Andersen Behavioral Model (ABM) developed by Ronald M. Andersen to investigate healthcare use and health outcomes [28]. The ABM includes predisposing variables, enabling variables, and need variables, and was used in the current study as a model to organize the variables by definition for analysis [28]. Variables were selected for this study on the basis of their perceived relevance to the topic and their availability in the dataset. The predisposing variables in this study were age, sex, race, and Hispanic status. The enabling variables in this study were schooling, employment, marriage, household income, and health insurance status. The need variables in this study were pain, comorbid conditions, general health, mental health, exercise, and smoking status [2,27].

Data analysis was conducted using Statistical Analysis System (SAS; v.9.4, SAS Institute Inc., Cary, NC, USA) software and utilized the SAS survey procedures “PROC SURVEY FREQ” and “PROC SURVEY LOGISTIC”, as is appropriate for complex survey data such as MEPS. The characteristics of US adults with arthritis who had limited physical functioning versus those who did not were compared using chi-squared tests. Three logistic regression models were developed to observe if and how the effect sizes changed as additional groups of variables were added. The first model tested the associations between predisposing variables and limited physical functioning. The second model tested associations between the predisposing and enabling variables and limited physical functioning. The third model tested the associations between the predisposing, enabling, and need variables and limited physical functioning. No limited physical functioning served as the reference group in all models. An alpha value of 0.05 was selected a priori. MEPS variables to account for clusters and strata within the data, and to calculate weighted population-based data values, were used.

## 3. Results

This study included 5102 US adults with arthritis, reflecting an estimated weighted population of 64,136,870 US adults with arthritis. This was stratified by limited physical functioning status, where 1997 US adults had limited physical functioning and 3105 did not have limited physical functioning. These samples provided an estimated weighted population of 21,745,191 US adults with arthritis who had limited physical functioning and 42,391,678 who did not have limited physical functioning.

The study participants were typically older (e.g., age groups ≥ 70 or 60–69), female, white, non-Hispanic, had more than high school education, unemployed, married, had high household income, had private health insurance, had little pain, had 2–3 comorbid conditions, had excellent/very good general health, had excellent/very good mental health, did not exercise, and did not smoke. Significant differences were present among groups for all variables (*p* < 0.05) apart from Hispanic status (*p* = 0.31; Table 1).

In the final multivariable logistic regression model, age ≥ 70 and ages 60–69 (vs. 18–49 years) was associated with higher odds of the person stating they had limited physical functioning, as was female (vs. male) sex. High school or less (vs. more than high school), being employed (vs. unemployed), and being married (vs. not married) were all associated with lower odds of the person stating they had limited physical functioning. Having quite a bit/extreme or moderate (vs. little) pain and having 6+ or 4–5 (vs. 0–1) comorbid conditions were all associated with higher odds of the person stating they had limited physical functioning; while having excellent/very good or good (vs. poor) general health and exercise (vs. no exercise) were all associated with lower odds of the person reporting they had limited physical functioning (Table 2).

## 4. Discussion

Among the predisposing variables, age ≥ 70 and ages 60–69 (vs. 18–49 years) were associated with higher odds of the person stating they had limited physical functioning. Data from several years of the NHIS (2010–2019) found the proportion of US adults with limited physical functioning increased with age, with the greatest proportion among those aged over 65 relative to the 18–64 age group [29]. In addition, arthritis typically increases with age, affecting approximately 29% of 50–64-year-olds, 44% of 65–74-year-olds, and 54% of adults 75 years and older according to recent (2022) NHIS data [14]. The association between age and limited physical functioning in the current study is therefore supported by existing knowledge yet understates the importance of remembering that limited physical functioning can occur at any age among US adults with arthritis.

Female (vs. male) sex was associated with lower odds of the person stating they had limited physical functioning in the current study. This finding parallels that of recent (2022) NHIS data that reported arthritis was higher among females (22%) than males (16%) [14], and data from 14 countries (US, Switzerland, Sweden, Spain, Netherlands, Italy, Ireland, Greece, Germany, France, England, Denmark, Belgium, and Austria) that reported that the prevalence of limited physical functioning was higher among women than men [30]. Although white vs. other races was associated with limited physical functioning in the initial model, this relationship was not observed in the final model. Previous studies have identified racial/ethnic differences in limited physical functioning among samples of the US population, with non-Hispanic and white people often having better outcomes than Hispanic people or people of other races [31,32,33,34]. It is encouraging that there was no association of race/ethnicity in the fully adjusted model in this study, which used nationally representative data that were collected more recently than the previous studies cited [31,32,33,34]. However, additional studies are needed to confirm these findings, and ongoing efforts are needed to monitor and address any racial and ethnic disparities among US adults with arthritis.

Among the enabling variables, high school or less (vs. more than high school), being employed (vs. unemployed), and being married (vs. not married) were associated with lower odds of the person stating they had limited physical functioning. The association between education status and limited physical functioning is an interesting one. Having limited physical functioning is thought to reduce a person’s educational attainment [1], while greater educational attainment has been found to be protective against arthritis [35]. This is important, given the prevalence of juvenile arthritis cases in the US [36], and suggests that greater efforts are needed to help reduce any impact of arthritis on educational attainment. As for employment, it is possible that US adults with arthritis and limited physical functioning are unable to work due to their condition and/or have retired from the workforce. This might explain the lower odds associated with employment and limited physical functioning among this population of US adults with arthritis. Among young adults (18–29-year-olds), 2009–2014 data from the NHIS found that those with arthritis had greater limited physical functioning than those without, and in multivariable models, those with arthritis were associated with lower odds of participation in education yet higher odds of employment [37]. The influence of marriage on limited physical functioning is unclear in the literature, though one article reported that spousal associations exist for limited physical functioning and can influence the trajectory of a person’s health in later life [38]. Therefore, additional research to better understand the impact of enabling variables on limited physical functioning among US adults with arthritis (potentially for different age groups or at different life points) may be warranted.

Although household income and health insurance were both associated with limited physical functioning in the second model, this relationship was not observed in the final model. Financial resources such as household income and health insurance are important factors for people with chronic conditions such as arthritis and limited physical functioning, particularly as it relates to healthcare costs and utilization of services. Research among Medicare Advantage beneficiaries showed that those with limited physical functioning who did not have assistance with their medicines had a higher likelihood of reporting cost-associated non-adherence to treatment [39]. Older community-dwelling adults with limited physical functioning have been shown to have high healthcare costs, from the health-system, personal, and societal perspectives [40]. In addition, a study using 2020 MEPS data reported that older US adults with pain and limited physical functioning were associated with 57% greater overall costs and 54% greater prescribed medicine costs relative to those with no limited physical functioning [41]. Furthermore, a 2011 MEPS analysis found that US individuals with arthritis or joint pain paid $1638 more in healthcare costs than those without arthritis or joint pain [42]. With reference to the analysis in Model 2 that accounted for predisposing and enabling factors, it is logical that US adults with arthritis were associated with higher odds of limited physical functioning if they had lower household income or private/public health insurance. Those with limited physical functioning may be less able to work, and therefore have a lower household income. Further investigation is needed to understand why these associations were no longer significant when additional need variables were accounted for in the model.

Among the need variables, having quite a bit/extreme or moderate (vs. little) pain was correlated with higher odds of the person reporting they had limited physical functioning. This is unsurprising, given that arthritis is a painful condition [12] and that limited physical functioning may be the cause of pain or a consequence of pain. Previous analyses of MEPS data have found a similar relationship between higher levels of pain and the presence of limited physical functioning [7,8,43].

Having 6+ or 4–5 (vs. 0–1) comorbid conditions was correlated with greater odds of the person stating they had limited physical functioning. This is again unsurprising, given that we might assume those with more comorbid conditions are less likely to be healthy and more likely to have a disability or limited physical functioning as a result of their comorbidities. This finding is similar to that found in the Canadian Longitudinal Study on Aging that reported that increasing numbers of comorbid conditions were related to greater odds of having limited physical functioning [44].

Meanwhile, having excellent/very good or good (vs. poor) general health and exercise (vs. no exercise) were both correlated with lower odds of the person reporting they had limited physical functioning in the current study. These are logical results that may be explained by the healthy user effect, whereby healthier people have fewer disabilities, and regular exercise helps prevent issues such as limited physical functioning in future. It is also likely that those with limited physical functioning would consider their health to be poorer than others without such a limitation and are unlikely to be able to do regular exercise. A cohort study of older adults between 1998 and 2000 reported that lack of frequent exercise was the most important factor in determining the likelihood of limited physical functioning. Lack of regular exercise is a modifiable risk factor to lower the risk of developing limited physical functioning in later life [25].

There was no association between mental health and smoking status and having limited physical functioning. A 2017 MEPS analysis of older US adults with pain also found no statistical association between mental health or smoking status and limited physical functioning [7]. Interestingly, the Canadian Longitudinal Study on Aging mentioned earlier found the correlation between comorbid conditions and limited physical functioning was stronger when the list of possible comorbid conditions included mental health conditions as compared with when the list of possible comorbid conditions did not include mental health conditions, which demonstrates the influence of mental health conditions on limited physical functioning [44]. Meanwhile, another MEPS analysis found that few demographic characteristics, including age and education level, were associated with mental health status among US adults with arthritis [45]. Given the increased emphasis on mental health care in recent years, it could be argued that the relationship between mental health and limited physical functioning in adults with arthritis should be reassessed in future. The lack of an association between smoking status and limited physical functioning is an interesting finding, given that one might expect smokers to have higher odds of limited physical functioning, particularly later in life due to the consequences of smoking. In addition, smoking is a recognized risk factor for arthritis [46]. Regardless, there is still merit in encouraging smoking cessation among this population to prevent any further health issues or disabilities [47].

One important limitation of this cross-sectional database study is the inability to demonstrate a cause-and-effect relationship of each of the predisposing, enabling, and need variables with limited physical functioning. Instead, only a statistical correlation can be concluded from this analysis. Another limitation is the potential for recall bias from the self-reported MEPS data, as well as people overestimating or underestimating their level of physical functioning.

## 5. Conclusions

This cross-sectional database study using a nationally representative sample of 2021 MEPS data has identified and described the predisposing, enabling, and need variables of interest in US adults with arthritis and their association with limited physical functioning. While there is existing literature or logical interpretations that explain many of the results, additional work may be needed to explore the others in greater detail.

## Figures and Tables

**Table 1:** Characteristics of United States adults in the weighted study population, organized by limited physical functioning status.

Variable	Total Wtd % [95% CI]	Limited Physical Functioning Wtd % [95% CI]	No Limited Physical Functioning Wtd % [95% CI]	*p*-Value
**Predisposing variables**				
Age (years)				<0.001
≥70	36.6 [34.7–38.4]	44.0 [40.9–47.0]	32.7 [30.5–35.0]	
60–69	28.0 [26.3–29.7]	28.9 [26.1–31.7]	27.5 [25.4–29.6]	
50–59	19.4 [17.8–21.1]	16.2 [13.6–18.7]	21.1 [19.1–23.2]	
18–49	16.0 [14.4–17.7]	11.0 [8.9–13.1]	18.6 [16.4–20.8]	
Sex				0.001
Female	60.6 [59.0–62.2]	64.4 [61.6–67.2]	58.7 [56.7–60.6]	
Male	39.4 [37.8–41.0]	35.6 [32.8–38.4]	41.3 [39.4–43.3]	
Race				0.03
White	80.7 [78.6–82.7]	78.3 [75.4–81.2]	81.9 [79.6–84.3]	
Other	19.3 [17.3–21.4]	21.7 [18.8–24.6]	18.1 [15.7–20.4]	
Hispanic				0.31
Yes	9.2 [7.6–10.7]	8.5 [6.6–10.3]	9.5 [7.7–11.3]	
No	90.8 [89.3–92.4]	91.5 [89.7–93.4]	90.5 [88.7–92.3]	
**Enabling variables**				
Schooling				<0.01
High school or less	42.0 [40.1–43.9]	46.8 [43.8–49.8]	39.6 [37.2–42.0]	
More than high school	58.0 [56.1–59.9]	53.2 [50.2–56.2]	60.4 [58.0–62.8]	
Employment				<0.001
Employed	41.0 [39.1–42.8]	23.6 [21.2–26.0]	49.9 [47.3–52.4]	
Unemployed	59.0 [57.2–60.9]	76.4 [74.0–78.8]	50.1 [47.6–52.7]	
Marriage				<0.001
Married	53.4 [51.5–55.4]	40.8 [37.6–44.0]	59.9 [57.7–62.2]	
Not married	46.6 [44.6–48.5]	59.2 [56.0–62.4]	40.1 [37.8–42.3]	
Household income				<0.001
Low	30.7 [28.7–32.7]	44.8 [41.7–47.8]	23.5 [21.3–25.8]	
Mid	27.2 [25.2–29.2]	26.0 [23.4–28.7]	27.9 [25.4–30.3]	
High	42.0 [39.7–44.4]	29.2 [26.1–32.3]	48.6 [45.7–51.5]	
Health insurance				<0.001
Private	58.2 [56.2–60.3]	45.2 [42.1–48.2]	65.0 [62.5–67.4]	
Public	39.5 [37.6–41.4]	54.0 [50.9–57.0]	32.1 [30.0–34.3]	
None	2.2 [1.5–3.0]	0.8 [0.2–1.5]	2.9 [1.9–4.0]	
Need variables				
Pain				<0.001
Quite a bit/extreme	29.3 [27.1–31.4]	49.1 [46.0–52.2]	13.6 [11.5–15.6]	
Moderate	21.8 [19.8–23.9]	22.8 [20.2–25.3]	21.1 [18.5–23.8]	
Little	48.9 [46.6–51.2]	28.1 [25.4–30.9]	65.3 [62.4–68.2]	
Comorbid conditions				<0.001
6+	6.4 [5.5–7.3]	12.6 [10.7–14.5]	3.2 [2.4–4.0]	
4–5	17.6 [16.3–18.9]	26.3 [23.9–28.8]	13.1 [11.7–14.5]	
2–3	40.0 [38.3–41.7]	38.2 [35.5–41.0]	40.9 [38.6–43.1]	
0–1	36.1 [34.3–37.9]	22.9 [20.3–25.4]	42.8 [40.5–45.2]	
General health				<0.001
Excellent/very good	39.4 [37.4–41.4]	19.7 [17.2–22.2]	49.5 [47.1–51.9]	
Good	35.0 [33.2–36.9]	34.9 [32.3–37.6]	35.1 [32.8–37.4]	
Poor	25.6 [24.0–27.1]	45.4 [42.3–48.4]	15.4 [13.7–17.1]	
Mental health				<0.001
Excellent/very good	50.1 [48.0–52.3]	38.0 [35.1–40.9]	56.3 [53.8–58.9]	
Good	35.4 [33.4–37.4]	37.6 [34.6–40.5]	34.3 [31.9–36.8]	
Poor	14.5 [13.2–15.7]	24.5 [21.7–27.2]	9.3 [8.1–10.6]	
Exercise				<0.001
Yes	46.6 [44.7–48.6]	32.3 [29.6–35.0]	54.0 [51.4–56.6]	
No	53.4 [51.4–55.3]	67.7 [65.0–70.4]	46.0 [43.4–48.6]	
Smoking				<0.001
Current smoker	13.7 [12.3–15.0]	17.1 [14.7–19.5]	11.9 [10.2–13.6]	
Nonsmoker	86.3 [85.0–87.7]	82.9 [80.5–85.3]	88.1 [86.4–89.8]	

Wtd = weighted; CI = confidence interval. The difference between the groups with limited physical functioning and no limited physical functioning for each variable was assessed using the chi-squared test.

**Table 2:** Associations of the variables with limited physical functioning among United States adults with arthritis.

Variable	Predisposing Variables (Model 1) OR [95% CI]	Predisposing and Enabling Variables (Model 2) OR [95% CI]	Predisposing, Enabling, and Need Variables (Model 3) OR [95% CI]
**Predisposing variables:**			
Age: ≥70 years vs. 18–49 years	2.35 [1.78–3.09] *	1.32 [0.96–1.82]	1.87 [1.23–2.86] *
Age: 60–69 years vs. 18–49 years	1.82 [1.36–2.43] *	1.35 [0.99–1.85]	1.64 [1.07–2.51] *
Age: 50–59 years vs. 18–49 years	1.33 [0.98–1.81]	1.26 [0.90–1.75]	1.09 [0.70–1.70]
Sex: female vs. male	1.28 [1.10–1.49] *	1.12 [0.95–1.33]	1.28 [1.04–1.56] *
Race: White vs. other	0.76 [0.62–0.93] *	0.95 [0.76–1.18]	1.03 [0.78–1.36]
Hispanic: yes vs. no	0.95 [0.73–1.22]	0.79 [0.60–1.03]	0.79 [0.52–1.19]
**Enabling variables:**			
Schooling: high school or less vs. more than high school		0.93 [0.79–1.11]	0.73 [0.59–0.91] *
Employment: employed vs. unemployed		0.44 [0.37–0.54] *	0.72 [0.55–0.95] *
Marriage: married vs. not married		0.59 [0.50–0.70] *	0.51 [0.40–0.64] *
Household income: low vs. mid		1.94 [1.50–2.50] *	1.07 [0.76–1.51]
Household income: low vs. high		1.29 [1.03–1.62] *	0.92 [0.67–1.27]
Health insurance: private vs. none		3.12 [1.30–7.50] *	2.04 [0.65–6.38]
Health insurance: public vs. none		4.16 [1.74–9.97] *	2.28 [0.72–7.20]
**Need variables:**			
Pain: quite a bit/extreme vs. little			5.19 [3.98–6.77] *
Pain: moderate vs. little			2.09 [1.64–2.66] *
Comorbid conditions: 6+ vs. 0–1			2.23 [1.45–3.42] *
Comorbid conditions: 4–5 vs. 0–1			1.65 [1.20–2.26] *
Comorbid conditions: 2–3 vs. 0–1			1.05 [0.80–1.37]
General health: Excellent/very good vs. poor			0.43 [0.30–0.60] *
General health: good vs. poor			0.66 [0.51–0.85] *
Mental health: Excellent/very good vs. poor			0.95 [0.66–1.36]
Mental health: good vs. poor			0.92 [0.65–1.31]
Exercise: yes vs. no			0.67 [0.54–0.83] *
Smoking: current smoker yes vs. non-smoker			1.38 [0.97–1.96]

OR = odds ratio; CI = confidence interval. * = statistical significance. C-statistics: Model 1 = 0.56; Model 2 = 0.70; Model 3 = 0.79. All Wald statistics < 0.001.

## Data Availability

No new data were created or analyzed in this study. Data sharing is not applicable to this article.
